# Are We Ready for Detecting α-Synuclein Prone to Aggregation in Patients? The Case of “Protein-Misfolding Cyclic Amplification” and “Real-Time Quaking-Induced Conversion” as Diagnostic Tools

**DOI:** 10.3389/fneur.2018.00415

**Published:** 2018-06-06

**Authors:** Silvia Paciotti, Giovanni Bellomo, Leonardo Gatticchi, Lucilla Parnetti

**Affiliations:** ^1^Department of Experimental Medicine, University of Perugia, Perugia, Italy; ^2^Magnetic Resonance Center (CERM), University of Florence, Sesto Fiorentino, Italy; ^3^Laboratory of Clinical Neurochemistry, Department of Medicine, University of Perugia, Perugia, Italy

**Keywords:** PMCA, RT-QuIC, α-synuclein, synucleinopathies, early diagnosis

## Abstract

The accumulation and deposition of α-synuclein aggregates in brain tissue is the main event in the pathogenesis of different neurodegenerative disorders grouped under the term of synucleinopathies. They include Parkinson's disease, dementia with Lewy bodies and multiple system atrophy. To date, the diagnosis of any of these disorders mainly relies on the recognition of clinical symptoms, when the neurodegeneration is already in an advanced phase. In the last years, several efforts have been carried out to develop new diagnostic tools for early diagnosis of synucleinopathies, with special interest to Parkinson's disease. The Protein-Misfolding Cyclic Amplification (PMCA) and the Real-Time Quaking-Induced Conversion (RT-QuIC) are ultrasensitive protein amplification assays for the detection of misfolded protein aggregates. Starting from the successful application in the diagnosis of human prion diseases, these techniques were recently tested for the detection of misfolded α-synuclein in brain homogenates and cerebrospinal fluid samples of patients affected by synucleinopathies. So far, only a few studies on a limited number of samples have been performed to test PMCA and RT-QuIC diagnostic reliability. Neverthless, these assays have shown very high sensitivity and specificity in detecting synucleinopathies even at the pre-clinical stage. Despite the application of PMCA and RT-QuIC for α-synuclein detection in biological fluids is very recent, these techniques seem to have the potential for identifying subjects that will be likely to develop synucleinopathies.

## Introduction

Protein-Misfolding Cyclic Amplification (PMCA) and Real-Time Quaking-Induced Conversion (RT-QuIC) represent two ultrasensitive protein amplification methods for detecting pathological protein aggregates in patients affected by protein misfolding disorders (1–3). PMCA and RT-QuIC are assays conceptually similar to a polymerase chain reaction (PCR): a template (protein aggregate) grows at the expense of a substrate (protein monomer) in a cyclic reaction characterized by a growth step followed by an increase in template units. Currently, the need of specific and sensitive early diagnostic tools for synucleinopathies points out the attention on novel approaches. Since α-synuclein (α-syn) follows aggregation mechanisms similar to PrP, PMCA and RT-QuIC assays were tested for the detection of misfolded α-syn in samples of patients affected by synucleinopathies (4–9).

A critical analysis on PMCA and RT-QuIC available data and protocols could help in evaluating whether these techniques could be suitable for the detection of α-syn aggregates in body fluids with high sensitivity and specificity, hopefully at a preclinical stage (4–7). The aim of this review is to provide an overview on existing data on PMCA and RT-QuIC assays, and their possible application for the diagnosis of synucleinopathies.

### PMCA and RT-QuIC: a brief history

The first PMCA protocol was developed by Soto's group in 2001 to detect the misfolded prion protein (PrP^Sc^) (10). The multiplication of the template units was performed by sonication followed by an incubation phase to let the aggregates grow. These steps were repeated several times in a cyclic process to allow the detection of the misfolded proteins in the samples [e.g., brain homogenates (BH), urine, blood, cerebrospinal fluid (CSF) and saliva]; at the end of the process, proteinase K (PK) digestion and western blot (WB) analysis were used to characterize and recognize the presence of pathological aggregates. The PMCA technique was tested in the subsequent years on biological samples coming from animals and patients affected by transmissible spongiform encephalopathy (11, 12). Atarashi et al., taking advantage on PMCA method, developed the QuIC assay by introducing some variants in the protocol (2, 13, 14). In the QuIC, the PrP^C^ substrate coming from hamsters BH was replaced by recombinant PrP^C^ and sonication was replaced with a vigorous intermittent shaking which promoted seeded aggregation of the monomeric substrate (13). Moreover, the WB analysis was substituted by a real-time monitoring (hence the name RT-QuIC) of the fluorescence emitted by the amyloid-sensitive Thioflavin-T dye (ThT) during the aggregation process (2, 14).

Although PMCA and RT-QuIC are both highly sensitive and specific assays, they showed different accuracy in detecting sporadic and variant Creutzfeldt-Jakob disease (CJD), also depending on the nature of the biological samples analyzed (15–17). The success of RT-QuIC in diagnosing prion diseases, led to test this assay for the detection of synucleinopathies (5, 6, 7). For this purpose, an αSyn-PMCA assay, methodologically very similar to a RT-QuIC was also developed by Soto's group (4).

### α-Synuclein and synucleinopathies

α-syn is a small protein (~14 kDa) largely present in the central nervous system at the pre-synaptic neuronal terminals (18, 19). Although α-syn was discovered almost 30 years ago, the physiological role carried out by this protein is not completely understood. It seems to be involved in the regulation of neurotransmitter release, synaptic plasticity and vesicle trafficking, in brain lipid metabolism, remodeling of the membranes, formation of membrane channels, and modification of their activity (20–23).

α-syn is composed of 140 amino acids and it is characterized by 3 distinct regions: N-terminal, central and C-terminal regions. The N-terminus (1–60 residues) contains seven highly conserved hexameric motifs, which form an amphipathic α-helix structure typical of the lipid binding domain of apolipoproteins (24), while the C-terminus (96–140 residues) contains multiple phosphorylation sites and it is enriched in acidic residues. The central domain of α-syn (61–95 residues), known as the non-amyloid-component (NAC), is highly aggregation-prone and plays a key role in cytotoxicity of α-syn (25–27).

At cellular level, α-syn is predominantly present as unfolded soluble monomer with not well-defined secondary or tertiary structures (28–30). Nevertheless, several factors like post-translational modifications (31–33), oxidative stress (28), fatty acids concentration (34–36), proteolysis (37, 38), phospholipids and metal ions (28, 29) can promote the misfolding of α-syn with the consequent formation of oligomers and amyloid-like fibrils (39, 40). α-syn amyloid-like fibrils are composed of several protofilaments containing cross β-sheet secondary structure in which individual β-strands run perpendicular to the fiber axis (41, 42). The α-syn aggregation kinetics is similar to that of the Aβ peptide (43, 44). It is characterized by an initial lag-phase which reflects the seed formation (nucleation phase) and a subsequent growth phase that culminates in a steady state (45).

Aggregated α-syn is involved in the pathogenesis of different neurodegenerative disorders known as synucleinopathies (46–48), which include Parkinson's disease (PD) (49), dementia with Lewy bodies (DLB) (50) and multiple system atrophy (MSA) (51). Fibrillary α-syn is the major constituent of Lewy bodies (LBs) and Lewy neurites (LNs), which represent the main histopathological hallmarks of PD and DLB (46, 47). Differently, in MSA, aggregated α-syn is found in oligodendrocytes as glial cytoplasmic inclusions (48).

The diagnostic value of α-syn as biomarker of synucleinopathies has been extensively investigated (52–56). Several studies have been performed to measure the levels of α-syn species (total, oligomeric and phosphorylated) in body fluids using different techniques: ELISA (57–60), multiplex immunoassays (61, 62), and Förster's resonance energy transfer (63). The heterogeneity of the applied methods partly justifies some ambiguous outcome obtained so far from the available studies. Furthermore, the lower concentration of the oligomeric/fibrillary α-syn species with respect to the monomeric α-syn form and the complexity to develop selective antibodies having high affinity and avidity to the misfolded α-syn species, make it difficult the detection of these species by using the most common antibodies-based assays (52, 64, 65).

The detection of pathogenic aggregates could help in diagnosis, both in terms of specificity and timeliness of diagnosis, since α-syn aggregation is an early phenomenon preceding the onset of clinical symptoms (66).

## RT-QuIC and PMCA assays: basic concepts

**Figure 1:**
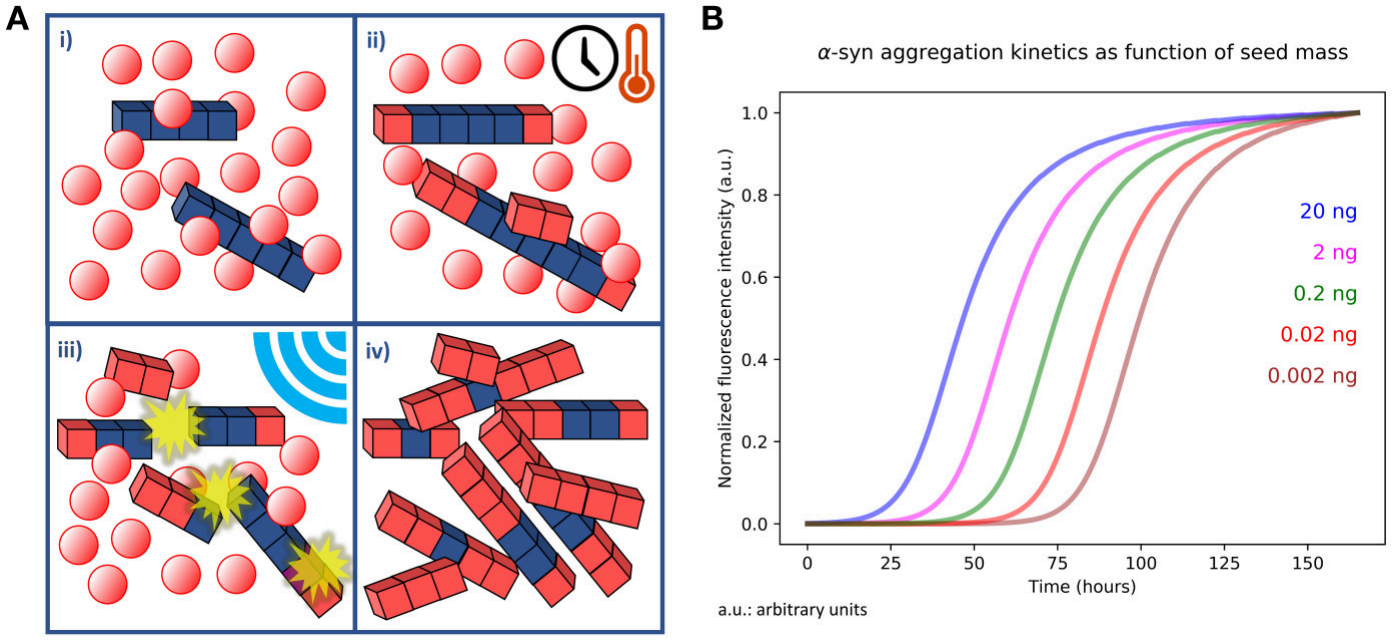
**(A)** Outline of PMCA and RT-QuIC kinetic assays. (i) An aliquot of tissue homogenate or a biological fluid containing a quantity of aggregates (blue cubes) is dissolved in a buffer containing abundant monomer in solution (red spheres). (ii) The sample is incubated for a defined time at specific temperature. In this phase the preformed aggregates undergo polymerization at their extremities and catalyze the formation of new nuclei on their surfaces. Monomers that undergo misfolding are depicted as red cubes. (iii) The number of available points for polymerization is increased by performing sonication or shaking of the sample, thus fragmenting the fibrils grown in the previous step. The steps (ii) and (iii) are repeated several times. (iv) At the end of the procedure the initial quantity of misfolded and aggregated protein is exponentially amplified at the expense of the monomer present in solution. **(B)** Simulation of a PMCA or RT-QuIC experiment. The simulation was performed by integrating differential equations describing polymerization, secondary catalyzed nucleation and fragmentation kinetics in presence of different quantities (20, 2, 0.2, 0.02, and 0.002 ng) of preformed aggregates (seeds). The simulation consisted in cycles of 30 min in which fragmentation kinetics was kept active only for 1 min (shaking) and turned off for 29 min (incubation). The cycles were repeated for a total time of 150 h. Normalized fluorescence intensity was calculated by considering it proportional to the total mass of fibrillary aggregates formed at a certain time.

The RT-QuIC and PMCA techniques are based on the amplification of a preformed quantity of misfolded proteins present in biological fluids or tissue samples. Samples are incubated, at a defined temperature, in a buffer solution containing the monomeric substrate. Preformed aggregates (seeds) work as templates polymerizing at their extremities at the expense of the monomer (Figure 1A). By introducing a shaking/sonication step, the grown aggregates are then fragmented to generate more polymerization points (67). Incubation and fragmentation cycles are repeated multiple times to achieve an exponential amplification of the aggregates. Apart from the basic polymerization and fragmentation processes, also surface catalyzed nucleation should be considered in the aggregation kinetics (68). This mechanism consists in the formation of new nuclei of misfolded proteins on the surface of preformed fibrils and it has been recently proposed for PrP^Sc^ (69), Aβ peptides (68, 70), and α-syn (71).

In PMCA, WB analysis is used to detect the amplified PrP^Sc^ (10), while in the RT-QuIC and αSyn-PMCA the detection of the misfolded aggregates is performed by recording the fluorescence of the ThT dye. ThT fluorescence (excitation at 450 nm and emission at 480 nm) is enhanced upon binding to fibrils. Compared to WB, ThT fluorescence assay has the limitation to be sensitive only to fibrillary aggregates rich in cross-beta sheet motifs (72). However, ThT assay in multi-well plates has the advantage to be less time-consuming; moreover, the intermittent shaking can be directly performed inside fluorometers and thus easily automated. The recorded fluorescence of ThT in RT-QuIC and αSyn-PMCA is proportional to the mass of fibrillary aggregates present in the sample and its trend gives information about the aggregation kinetics of the monomer. Fluorescence acquisition allows mapping an aggregation curve describing a lag-phase (time with stationary fluorescence), an exponential phase (increase in fluorescence) and a plateau. A simulated example of an ideal output of a RT-QuIC experiment is shown in Figure 1B. The process produces sigmoid-like profiles (73, 74) whose lag-times, slopes and stationary points depend on the experimental conditions (temperature, shaking cycles and strength, pH, buffer, etc.). Particularly, the length of the lag-phase correlates to the amount of seeds in the samples (75). However, since the lag-phase is a threshold value established by the investigator, the t50, named the time necessary to reach the 50% of the maximum fluorescence, is often used as a quantitative and objective measurement of the amplification process. The approximate linear relation between the t50 (or the lag-time) and the logarithm of the seed quantities has been shown for different pathogenic proteins like PrP^sc^ (75) α-syn (4, 7, 76), Aβ1-40 (77), and Aβ1-42 (78). Sometimes, deviations from the ideal lineshape, like multiple inflection points or a decrease of the signal at the end of the reaction are present (6, 79). These abnormalities might be caused by sample heterogeneity (amyloids tend to form a suspension in aqueous solution) or by the entrapment of ThT in large aggregates, respectively (80). Thus, most of the authors prefers to define a lag-phase threshold, in which controls do not exhibit aggregation, while positive samples display an increase in fluorescence intensity that exceed the established threshold (e.g., 5–10 times higher than average baseline fluorescence) (4–7, 75, 81). Apart from the length of the lag-phase, Kang et al. (82) suggested that also differences in amyloid formation rate, ThT fluorescence maxima and integrated area under the curve show discrimination between seeded and unseeded samples, thus these features could be also suitable for αSyn-PMCA and RT-QuIC data analysis.

### Protocols

Several physical (temperature and sonication/shaking), chemical (ionic strength, pH, monomer concentration, detergents), and exogenous factors were described to affect α-syn aggregation kinetics (83, 84). The most recent implementations in PMCA and RT-QuIC protocols, specifically applied to the detection of α-syn aggregates for the diagnosis of synucleinopathies, are reported in Table 1 and discussed below.

**Table 1:** PMCA and RT-QuIC assays protocols.

**Assay**	**Sample**	**α-syn concentration (mg/ml)**	**Buffer**	**Sonication/shaking**	**Temperature (°C)**	**Beads^a^**	**Sample volume/final volume (μl)**	**Sample readout**	**Number of samples (cases/controls)**	**Sensitivity (%)**	**Specificity (%)**	**Detection limits^b^**	**References**
RT-QuIC	BH; CSF	0.1	40 mM phosphate (pH 8.0), 170 mM NaCl, 10 μM ThT, 0.0015% SDS (only for CSF)	1 min (400 rpm) 1 min rest (Sh)	42	6 beads (0.8 mm)	BH 2/100; CSF 15/100	ThT	29/31	93	100	BH 10^−6^; CSF 0.2 μl; α-syn fibrils 100 ag	(7)
RT-QuIC	BH	0.1–0.15	50 mM HEPES (pH 7.5), 10 μM ThT	40 s (432 rpm), 2 min rest (Sh)	40	–	BH (diluted) 5/100	ThT	13/2	–	100	BH 5 × 10^−6^	(6)
RT-QuIC	BH; CSF	0.1	100 mM phosphate (pH 8.2), 10 μM ThT	1 min (200 rpm), 14 min rest (Sh)	30	37 ± 3 mg (0.5 mm)	BH (1:20,000) 2/100; CSF 5–15/100	ThT	BH, 15/3; CSF, 102/35	92 DLB, 95 PD	100	–	(5)
PMCA	CSF	0.1 or 1	PBS (pH 7.4) or 100 mM PIPES (pH 6.5), 500 mM NaCl, 5 μM ThT	1 min (500 rpm), 29 min rest (Sh)	37	–	CSF 40/200	ThT	96/97	100 DLB, 88.5 PD, 80 MSA	94–96.9	150 amol	(4)
PMCA	BH	0.723	10 mM Tris (pH 7.5), 150 mM NaCl	10 s every 29 min 50 s (So)	37	10 beads (1 mm)	BH 1%	ThT	1/1	–	–	100 amol	(76)
PMCA	–	0.38 or 1.28	150 mM NaCl, 1% triton X-100, complete protease inhibitors in PBS	20 s every 30 min (So)	37	37 ± 3 mg (1 mm)	200	ThT; WB	–	–	–	–	(80)
PMCA	–	0.28	150 mM NaCl, 1% triton X-100	20 s every 29 min 40 s (So)	37	–	100	ThT; WB	–	–	–	–	(85)
PMCA	–	4.3	Physiological buffer (pH 7.4)	30 s every 10 min (x6) (So); 10 min (300 rpm, x6), 10 h constant, 10 min (x6) (Sh)	37	–	300	WB	–	–	–	–	(86)

*BH, brain homogenates; CSF, cerebrospinal fluid; SDS, sodium dodecyl sulfate; HEPES, (4-(2-hydroxyethyl)-1-piperazineethanesulfonic acid); ThT, Thioflavin T; PIPES, Piperazine-N,N'-bis(ethansulfonic acid); PBS, phosphate-buffered saline; So, sonication; Sh, shaking; WB, western blot; DLB, dementia with Lewy bodies; PD, Parkinson's disease; MSA, multiple system atrophy; ag, attograms; amol, attomoles*.

a *Number/mg of zirconia/silica beads per reaction*.

b *Detection limits as reported in the original papers*.

#### αSyn-PMCA and RT-QuIC substrate

*In vitro* aggregation assay usually requires large amounts of highly purified monomeric α-syn as reaction substrate for fibrils polymerization. Large quantities of recombinant α-syn are obtained by using *Escherichia coli* cultures. The expressed protein can be purified by different chromatographic procedures (7, 87–89). The purity of α-syn preparations can be evaluated by SDS-PAGE followed by silver staining and then confirmed by mass spectrometry. The quality of the initial α-syn monomer solution is a critical factor in determining the successful application of αSyn-PMCA and RT-QuIC techniques. α-syn monomer solution can be filtered with a 100 kDa cutoff filter device (4) in order to remove any preformed aggregates generated during the purification process. To use the optimal amount of substrate in αSyn-PMCA or RT-QuIC, the concentration of the purified α-syn is assessed by spectrophotometric measurement of absorbance at 280 nm (83, 86).

#### Temperature, pH, and buffer composition

Reaction temperature is one of the most well established factors driving α-syn aggregation (39, 90). Generally, in PMCA or RT-QuIC assay, the temperature is set at 37°C. Thirty-seven degree celsius is compatible with a balance between obtaining a short lag-phase, a stable elongation rate, and a minor evaporation of the sample. Similarly, the decrease of pH values toward the isoelectric point of α-syn (pI = 4.67) contributes to the neutralization of protein net charge, that enhances hydrophobicity and boosts the fibrillization process (91). Moreover, the rate of aggregates formation is enhanced by the increase in ionic strength of the reaction buffer (84).

Interestingly, Shahnawaz et al. reported an inhibitory effect of CSF for α-syn aggregation (4); the causes of this behavior are not yet well understood, although Padayachee et al. observed a similar effect also for Aβ (92). Shahnawaz et al. introduced the buffer with the best results in terms of α-syn aggregation timescales and sensitivity in the presence of CSF. By using this buffer, they were able to reduce significantly the lag-phase for positive samples and to decrease the detection sensitivity threshold to femtograms of preformed α-syn seeds. In addition, detergents can be added to reaction buffers to ensure the complete recovery of insoluble amorphous aggregates, together with soluble forms of α-syn fibrils when sonication rather than shaking is used (80, 85). Notably, generation of different species of α-syn aggregates is likely to be linked to different synucleinopathies (93, 94). The application of PMCA protocol allows to amplify brain-derived fibrils with conserved conformation of the original seed (85).

#### Incubation and agitation cycles

The introduction of incubation and agitation cycles played a key-role from the first implementation of PMCA to the last RT-QuIC. In the first version of PMCA (10) the sample was sonicated every hour (five pulses of 1 s each), while in the last RT-QuIC implementations, the sonication step has been replaced by automatic shaking in well plates. Particularly, in the works regarding α-syn, Jung et al. (85), Herva et al. (80), and Roostaee et al. (86) performed sonication on their samples for non-diagnostic applications (Table 1). Conversely, Fairfoul et al. (5), Shahnawaz et al. (4), Sano et al. (6), and Groveman et al. (7) applied the following cycles: 1 min shaking (200 rpm) with 14 min of incubation, 60 s shaking (432 rpm) with 2 min of incubation, 40 s shaking (500 rpm) with 29 min of incubation, and 1 min shaking (400 rpm) with 1 min of incubation, respectively. Shaking is one of the most important promoting factors of α-syn aggregation (83, 84). Nevertheless, it is also important to let the sample rest for some time to promote elongation phase: Herva et al. (80) noticed that alternating cycles of incubation and agitation produced a shorter lag-phase compared to continuous agitation. Furthermore, the addition of zirconia/silica beads to the samples increases the fragmentation and diffusion rates and improves the reproducibility of the assay (5, 80, 83).

## αSyn-PMCA and RT-QuIC studies in diagnostic cohorts

Currently, only a few studies have been performed to test the accuracy of PMCA and RT-QuIC as diagnostic tools for synucleinopathies. Groveman et al. performed RT-QuIC on CSF samples from 29 patients affected by synucleinopathies (12 PD and 17 DLB) and 31 non-synucleinopathy controls [including 16 patients affected by Alzheimer's disease (AD)] (7). Almost all synucleinopathy CSF samples (27 out of 29) gave positive RT-QuIC, whereas none of the non-synucleinopathy controls met the criteria to be considered positive (93% sensitivity and 100% specificity). In this work, an end-point dilution assay was also performed to quantify the RT-QuIC seeding activity in PD (*n* = 1) and DLB (*n* = 3) BH and DLB (*n* = 5) CSF samples by calculating the concentration of seeding activity units (SD_50_). The estimated SD_50_ was 10^5^-10^6^ per mg of brain tissue and 4–54 per 15 μl of CSF. These results indicate that CSF samples have seeding activities higher than the minimum detectable level of 1 SD_50_.

Fairfoul et al. tested the RT-QuIC technology on BH from patients affected by DLB, AD, CJD, and control subjects (5). None of the reactions seeded with BH from patients affected by CJD or AD as well as from control subjects gave positive results after 120 h from the beginning of the reaction. The same group analyzed CSF samples from the OPTIMA (Oxford Project to Investigate Memory and Ageing) cohort with the aim to investigate RT-QuIC sensitivity. The study included patients with clinically and neuropathologically confirmed diagnosis of DLB (*n* = 12), PD (*n* = 2), progressive supranuclear palsy (PSP) (*n* = 2), corticobasal degeneration (CBD) (*n* = 3), DLB with AD pathology (*n* = 17), AD with incidental LBs (*n* = 13), pure AD (*n* = 30), and controls (*n* = 20). DLB and PD patients were diagnosed with a 92 and 95% sensitivity, respectively, and with a specificity of 100%. A sensitivity of 65% was observed for patients affected by mixed AD/DLB pathology. None of the patients affected by PSP, CBD, or pure AD, resulted positive to RT-QuIC. A validation study was also carried out in CSF samples from 20 patients diagnosed as PD, 15 control subjects, and 3 subjects affected by rapid eye movement sleep behavior disorder (RBD), a condition at high risk of developing synucleinopathies. Out of 20, 19 PD patients resulted positive (sensitivity = 95%, specificity = 100%), whereas all controls were found negative. The three RBD also showed a positive RT-QuIC response, suggesting the suitability of this approach for early diagnosis.

Shahnawaz et al. used the αSyn-PMCA for detecting α-syn aggregates in CSF samples from different synucleinopathies (PD *n* = 76, DLB *n* = 10, MSA *n* = 10) and other miscellaneous neurological disorders (*n* = 97) including other neurodegenerative diseases not belonging to synucleinopathies (AD, frontotemporal dementia, PSP, ataxia) (4). Out of 76 PD patients, 67 (88%) resulted positive to αSyn-PMCA, whereas 61 out of 65 (94%) patients affected by other neurological disorders resulted negative. Notably, two samples, which were clinically diagnosed as PD after some years from sample collection, resulted positive, indicating the ability of αSyn-PMCA to identify patients even at the prodromal stage. All DLB patients and 8 out of 10 MSA cases were positive at αSyn-PMCA. Out of 14 AD patients, 5 showed positive results. This result might not be considered as false-positive, since α-syn inclusions are not rare in AD brain (95, 96). For this reason, sensitivity and specificity were calculated by excluding AD patients from the analysis. Sensitivity was 88.5% for PD, 100% for DLB and 80% for MSA. Specificity was 94%, reaching 97% when considering patients affected by neurological, but not neurodegenerative, disorders. In this study, the possible correlation between the disease severity and αSyn-PMCA kinetic parameters was also investigated in PD group. A significant negative correlation between the t50 in αSyn-PMCA and the Hoehn and Yahr scale was found. The reduction of the lag-phase suggests the presence of higher concentration of α-syn aggregates in CSF samples of advanced PD cases, thus allowing the monitoring of disease progression. However, these data need to be confirmed in a larger cohort.

Finally, Nishida's group investigated the presence of prion-like seeding of misfolded α-syn in brain samples from patients affected by DLB (*n* = 7), CJD (*n* = 3), Gerstmann-Sträussler-Scheinker disease (*n* = 1), pure AD (*n* = 2), and controls. They found positive results only in BH from DLB patients (6).

## Conclusion and future directions

The first trials of PMCA on PrP^Sc^ date back to 2001 but only recently the αSyn-PMCA and RT-QuIC techniques have been applied for the amplification and detection of aggregates of misfolded α-syn. The positive results obtained from different studies confirm that αSyn-PMCA and RT-QuIC are suitable assays for detecting α-syn aggregates in CSF samples. Furthermore, the high sensitivity and specificity of these techniques in detecting synucleinopathies, even at the pre-clinical stage, suggest their possible use as diagnostic tools. Although the combined analysis of α-syn aggregates with other CSF biomarkers (e.g., Aβ42, t-Tau and p-Tau) can be used in the cases of uncertain diagnosis (e.g., patients affected by mixed AD/DLB pathology), in-depth investigations are still necessary to perform a differential diagnosis among different synucleinopathies. The study of α-syn aggregation kinetics, the characterization of the fibrillary aggregate structure (e.g., by PK digestion, WB analysis, X-ray scattering and solid-state NMR) (42, 97–100), as well as the detection of other soluble or insoluble α-syn non-fibrillary aggregates might be suitable to this purpose (42, 94, 97, 98, 101).

Furthermore, the possibility to assess the SD_50_ in CSF samples, might be relevant for determining prognosis in patients even at the early stage of disease (4, 7). So far, αSyn-PMCA and RT-QuIC has been performed mainly in CSF samples; however, based on the encouraging results obtained in the diagnosis of prion disease in both human and animals (102–107), other more “easily accessible” biological fluids like blood, plasma, serum, urine and saliva, as well as peripheral tissues obtained from biopsies (e.g., nasal mucosa, gastrointestinal tract and skin) have the potential to be used as samples for the detection of misfolded α-syn.

Further developments are still needed to standardize operating procedures, decrease the duration of the assays, and increase their sensitivity. To this purpose, testing different shaking cycles and incubation temperatures will be crucial. The reproducibility of the method has also to be improved in order to uniform lag-times, maximum of fluorescence intensity and lineshapes among replicates.

In conclusion, αSyn-PMCA and RT-QuIC have the potential to be effective tools for the diagnosis of synucleinopathies. It will be exciting to follow the growth of scientific reports about this goal in the next future.

## Author contributions

SP, GB, LG, and LP wrote the paper. GB prepared illustrations. LP revised the text.

### Conflict of interest statement

The authors declare that the research was conducted in the absence of any commercial or financial relationships that could be construed as a potential conflict of interest.
